# A Review on the Conservation of South African Indigenous Poultry Breeds: A Focus on Semen Cryopreservation

**DOI:** 10.3390/ani15040529

**Published:** 2025-02-12

**Authors:** Rantloko Rolly Maapola, Jabulani Nkululeko Ngcobo, Khathutshelo Agree Nephawe, Tshimangadzo Lucky Nedambale, Fhulufhelo Vincent Ramukhithi

**Affiliations:** 1 Department of Animal Science, Tshwane University of Technology, Private Bag X608, Pretoria 0001, South Africa; ngcobojn@tut.ac.za (J.N.N.); nephaweka@tut.ac.za (K.A.N.); nedambaletl@tut.ac.za (T.L.N.); 2 Agricultural Research Council—Germplasm Conservation and Reproductive Biotechnologies, Private Bag X2, Irene 0062, South Africa; ramukhithif@arc.agric.za

**Keywords:** conservation, indigenous poultry breeds, cryoprotectants, cryopreservation

## Abstract

Understanding the genetic, physiological, and nutritional characteristics of native chickens in South Africa has been significantly hindered by studies over the last ten years. These chickens hold significant economic, social, and cultural importance for South African communities, particularly those marginalized. Despite their reputation for lower egg productivity, they are highly valued for their flavorful meat by consumers. Many local chicken ecotypes and breeds remain undocumented and in danger of going extinct, even though some have been classified. To tackle this issue, the Food and Agriculture Organization has launched an indigenous poultry conservation program. One crucial method employed is assisted reproductive biotechnologies such as cryopreservation, which serves as an ex situ conservation strategy for preserving the germplasm of endangered animals. In avian species, cryopreservation is particularly beneficial for the long-term storage of sperm cells, although it necessitates the use of cryoprotectants to shield sperm cells from cold shock during freezing. However, the use of cryoprotectants can lead to thermal shocks that may damage the sperm cell plasma membrane, potentially reducing viability and fertility. Furthermore, the membranes of avian sperm cells are highly polyunsaturated fatty acids, which can undergo lipid peroxidation (LPO) when reactive oxygen species (ROS) are present. This review focuses on current knowledge and the latest effective strategies for utilizing cryopreservation to conserve semen from indigenous poultry breeds.

## Simple Summary

Preserving genetic variety and safeguarding uncommon and threatened breeds of poultry in South Africa requires the conservation of native poultry breeds, especially through the cryopreservation of their semen. However, there are challenges associated with working with poultry semen, as cryopreservation can cause oxidative stress and damage to sperm membranes, reducing fertility rates. Despite these challenges, further research and investment in cryopreservation technologies could significantly improve the sustainability and success of South Africa’s poultry industry, particularly in the preservation and improvement of indigenous poultry breeds.

## 1. Introduction

South Africa has made strides in poverty reduction since 1994; however, the trajectory of poverty reduction reversed between 2011 and 2015, jeopardising previous gains [1]. A total of 18.2 million South Africans are estimated to be living below bread standard as of 2023, classified as those making less than ZAR 34.38 (USD 1.90) per day [2]. This is an increase of 162,859 per person over 2022, with more increases expected in the coming years. By 2030, it is estimated that over 19.1 million South Africans will be living on a maximum of ZAR 34.38 (USD 1.90) per day [2]. Income inequality remains a significant driver of poverty in this country.

Addressing food security is crucial amidst these challenges, with South Africa prioritising this goal by 2030 despite its rapidly growing population [3]. Indigenous chickens play a vital role in ensuring household food security in developing countries [4], contributing substantially to South Africa’s National Development Plan (NDP) 2030. Most studies indicate that agriculture contributes significantly to rural communities by providing employment, ensuring food and nutritional security, enhancing livelihood assets, and promoting gender equality, which may help to address food insecurity concerns raised [5,6,7]. In underdeveloped or developing countries like South Africa, the majority of smallholder farmers view agriculture as their full-time occupation [8]. For instance, local chickens hold significant economic, social, and cultural value, particularly for marginalised communities in Africa. These chickens provide both a source of income and protein for resource-limited households in developing countries [6]. They are often preferred over exotic breeds due to their succulent meat, less feed and fewer veterinary service requirements.

Indigenous breeds like Venda, Ovambo, Potchefstroom Koekoek, and Naked Neck are well adapted to harsh environments, exhibiting resistance to many local diseases and tolerating extreme temperatures, wet conditions, and drought. Moreover, they are relatively inexpensive to manage [9]. However, these chicken breeds are not well managed due to a lack of information regarding their reproductive performance [6]. Lack of information among rural farmers led to the extinction danger of these due to unsupervised crossbreeding and the introduction of exotic breeds to improve their colour patterns or body size. This extinction threat calls for immediate intervention to avoid extinction. Conserving them is crucial not only for safeguarding their diversity but also because, once they are extinct, they cannot be reversed or replaced [10].

The primary goal of gene conservation is to maintain superior and distinct genetic lines that are as pure as possible [11]. It is estimated that local chickens account for 80% of poultry production in Sub-Saharan Africa. While these figures underscore the need to boost poultry production, the quantity and quality of the products still require improvement. Therefore, the introduction of modern technologies, including reproductive technologies like cryopreservation, has become crucial for preserving genetic diversity and facilitating gene flow among populations.

Cryopreservation involves the preservation of tissues and cells by utilising certain small molecules to enter the cell and prevent dehydration and the formation of intracellular ice crystals, which can lead to cell death and damage to cell organelles during the process [12]. Furthermore, in assisted reproductive techniques, the freezing of semen is a valuable tool for genetic management, allowing for the storage of semen for future use. Therefore, the main purpose of freezing is to maintain the semen quality [13]. This method enables cells to be stored to prevent cell culture at all times, reduce genetic drift, and minimise morphological changes. Various steps such as dilution, cooling, freezing, and thawing are all involved during this process [14]. The conservation of germplasm from domestic and endangered avian species is essential. According to Donoghue and Wishart [15], in 1951, the first animals created from frozen, thawed sperm cells using glycerol were chickens. However, since then, there has been finite progress in the development of semen cryopreservation technology in the poultry industry.

In recent times, there has been limited commercial utilisation of frozen stored poultry sperm compared to other species. Due to the fact that chicken sperm has a longer tail, measuring about 90 μm, makes it more susceptible to damage during the freezing process, resulting in lower fertility rates [16]. Cryopreserving cockerel semen is currently the only practical method for conserving these sex cells, as yolk-laden eggs cannot be frozen [17]. However, cryopreserved cockerel sperm cells often exhibit highly variable fertility. The low tolerance of poultry sperm to cryopreservation can be attributed to their cellular and molecular characteristics. For instance, poultry sperm membranes contain less cytoplasm and mitochondria and a higher amount of polyunsaturated fatty acids than mammalian sperm, necessitating greater antioxidant protection during the freezing process [16]. Semen is primarily attributed to diminished fertility of the sperm cells post-freezing, a phenomenon commonly known as cold shock [18]. Cold shock arises when sperm cells are subjected to temperatures below their physiological range, leading to detrimental effects on cellular membranes and disruptions in metabolic function as a consequence of changes in membrane composition [19].

According to Ozkavukcu et al. [20], cryopreservation significantly reduces sperm cell motility and can cause damage to the plasma membrane, acrosome, and tail. Other researchers have reported varying percentages of frozen–thawed sperm cell motility: 22.7%; 45%; 25.71%; 29.06%; and 36.5% [21,22,23,24,25]. However, positive outcomes have been reported in recent studies using soya bean nanoparticles, and DMA extenders achieved sperm cell fertility rates of 47.30% and 41%, respectively, following cryopreservation of cockerel semen [26,27]. Nevertheless, there is still room for improvement in the cryopreservation of cockerel semen, as the obtained results remain low. Therefore, there is a need to improve the reproductive efficiency of breeding through the use of cryopreserved semen, which may improve post-thaw sperm cell motility [28]. This review will mainly focus on the conservation of South African indigenous poultry breeds using Assisted Reproductive Technology (ART) such as cryopreservation.

## 2. Conservation Strategies

Local chickens raised in smallholder low-input systems are vital genetic resources that need urgent conservation to counter threats from production pressures and the encroachment of commercial hybrids [29]. Alarmingly, approximately 33% of local chicken breeds are at risk of extinction, a challenge that extends beyond Southern African nations to developing countries globally [6]. Addressing this issue requires robust conservation programs that emphasise the value of indigenous chickens, which are increasingly recognised for their unique genetic traits in sustainable animal breeding. In South Africa, the significance of preserving chicken genetic resources has long been acknowledged. The South African Agricultural Research Council (ARC) has initiated a program dedicated to the genetic improvement and conservation of four local breeds: Naked Neck; Ovambo; Potchefstroom Koekoek; Boschveld; and Venda chickens [6,29]. These initiatives highlight a commitment to preserving local biodiversity while enhancing the resilience and sustainability of poultry production systems in South Africa.

**Figure 1:**
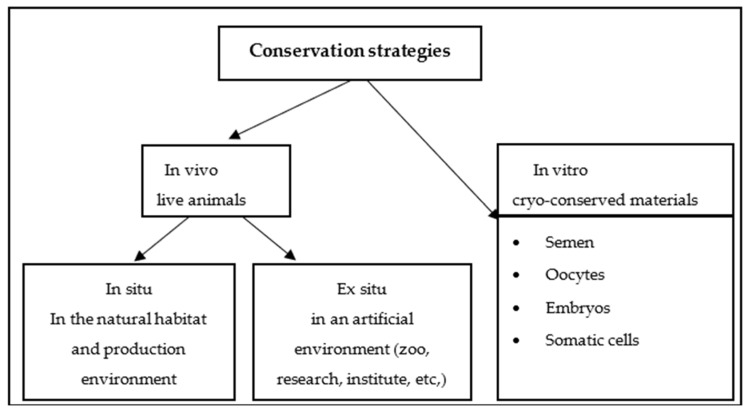
Schematic representation of conservation strategies. Source: [37].

Generally, conservation strategies for farm animal genetic resources (FAnGR) are categorised into three approaches: in situ; ex situ; and genetic resources conservation (Figure 1) [30,31]. In situ conservation focuses on preserving breeds in their natural habitats or on farms where they are traditionally raised [10]. Maintaining breeds in their native environments helps sustain genetic diversity and protect the unique characteristics of each breed [32]. Additionally, the periodic reproduction of in situ populations increases feeding costs and requires specialised facilities such as farms and poultry houses [33].

In contrast, ex situ conservation involves maintaining breeds outside their traditional environments through methods such as cryopreservation, which stores genetic material at low temperatures for future use [34]. This can also include establishing gene banks or controlled breeding programs to ensure the survival of endangered breeds [35]. Additionally, the periodic reproduction of in situ populations increases feeding costs and requires specialised facilities such as farms and poultry houses [33]. Ex situ conservation programs may face challenges in developing countries. Although it is possible to successfully cryopreserve the semen of various poultry species, including chickens, geese, ducks, and turkeys, the post-thaw fertility of poultry semen often lags behind that of other species, with notable differences among breeds, lines, or individuals [36]. Currently, ex situ conservation in poultry primarily focuses on commercially and industrially valuable breeds or lines through the collection of frozen semen [33]. For in vitro conservation of avian species, cryopreserving semen is currently the only practical method for conserving these sex cells, as yolk-laden eggs cannot be frozen.

## 3. An Overview of South African Indigenous Poultry Chickens

**Figure 2:**
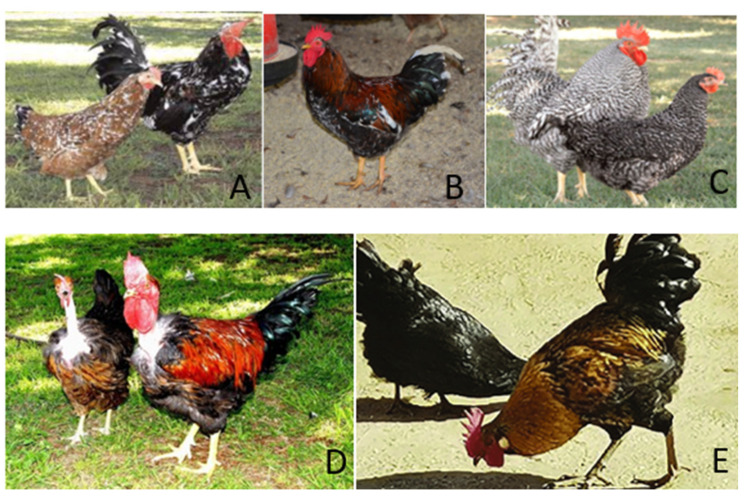
(**A**) Venda; (**B**) Boschveld; (**C**) Potchefstroom Koekoek; (**D**) Naked Neck; and (**E**) Ovambo. Source: [6].

Indigenous poultry breeds in South Africa play a vital role in the country’s agricultural landscape, particularly in rural communities. These breeds, such as the Venda, Naked Neck, Boschveld, Ovambo, and Potchefstroom Koekoek (Figure 2), are well-adapted to local conditions, exhibiting resilience to diseases and environmental stresses [38]. They are culturally significant, providing both food and income to resource-limited households [6]. Despite their advantages, these indigenous breeds face challenges from crossbreeding with exotic varieties and declining genetic diversity. Efforts to conserve and promote these breeds are essential for maintaining agricultural sustainability and food security in South Africa.

### 3.1. Venda

The Venda chicken breed was initially documented in 1979 by veterinarian Dr. Naas Coetzee in Venda village, situated in the Limpopo province. It was named after the area where it was found, which is located in Limpopo [9]. Subsequently, chickens of the Venda breed were also identified in Qwa-Qwa in the Free State and the Southern Cape. The Venda breed is distinguished by its multi-coloured appearance, with black, white, and red being the predominant colours [6]. Furthermore, this breed is relatively light and has soft feathers. The Venda breed is regarded as large in comparison with other indigenous breeds. It also produces tinted eggs of good size, and the hens are known for being broody and good mothers [9]. This breed caught the attention of Dr. Coetzee due to its ability to resist disease and have low management costs.

### 3.2. Boschveld

It is recognised that Boschveld chickens are a hybrid indigenous breed. Michael Bosch, a farmer in South Africa, made this discovery. The main reason he developed these chickens specifically was to solve the tick problem he was having with his cattle. In an attempt to rid his cattle of ticks at watering holes, the farmer raised a free-range breed of chicken. In 1998, they were bred at the Mantsole ranch in the South African region of Limpopo. Venda, Ovambo, and Matabele indigenous chickens were the three types of free-range breeds that were crossed to create this breed [39]. It is claimed that the Boschveld chicken inherited 25% of the Ovambo breed’s traits, 50% of the Venda breed’s traits, and 25% of the Matabele breed’s traits [6].

The feathers of Boschveld hens appear to be blended with white, giving them a light brown colouring. They are highly renowned for their alertness and hardiness, which contribute to their outstanding survival abilities in protecting themselves from predators and resistance to illnesses that affect poultry [40]. Like other roadrunners, this breed is renowned for its capacity to procreate in hostile environments and for being able to reproduce on its own. Boschveld cocks are renowned for being extremely powerful, aggressive, and nearly noble. The hens are fantastic free-range chickens with their chicks; they are equally robust and extremely fruitful [6].

### 3.3. Potchefstroom Koekoek

The Potchefstroom Koekoek chicken breed was developed in the 1950s at Potchefstroom Agricultural College in South Africa by Chris Marais. It is a free-range, dual-purpose breed that is suitable for both meat and egg production [9]. The goal of the breed’s founder was to create a chicken breed that would thrive in the Southern African climate and conditions. The Potchefstroom Koekoek is a cross between the Black Austrolorp and white Leghorn breeds, with a strain of the Barred Plymouth Rock breed added as well [9,41].

The feathers of Potchefstroom Koekoek chickens are striped in black and white, with unique patterns on both the hens and roosters. They have strong maternal instincts and are good brooders. At 16 weeks of age, roosters in this breed weigh approximately 1.84 kg, while hens weigh approximately 1.4 kg. They have soft feathers and are relatively hefty. The chickens can lay an average of 198 eggs annually once they reach sexual maturity, which occurs after 130 days. The weight of these eggs is 55.78 g on average. The hens have a 78% hatchability rate and are renowned for being broody and good watchers [6]. The Potchefstroom Koekoek’s ability to forage for its own food makes it an excellent bird for free-ranging and is particularly well-suited for smallholder farmers.

### 3.4. Naked Neck

It is thought that the South African Naked Neck chicken originated in Asia, more specifically in Malaysia, where it was bred for cockfighting for a very long time. There are two varieties of Naked Neck chickens: the purebred variety has a neck that is entirely devoid of feathers, while the non-purebred kind has a tassel on the front of the neck [6]. Although this breed has a distinctive appearance, it is not widely recognised as an exhibition bird. It is a multipurpose chicken that may be raised for both meat and eggs.

Large wattles and a single red comb characterise Naked Neck chickens. They have crimson earlobes and reddish–bay eyes. They are excellent foragers and have a strong immune system, making them resistant to most diseases. According to Islam and Nishibori [42] and Sharifi et al. [43], the featherless neck characteristic is influenced by an autosomal dominant gene (Na) with incomplete dominance. As the breed approaches maturity, this gene causes the skin on the neck to become plain and reddish. In the heterozygote state (Nana), a small cluster of feathers can be seen in the ventral section of the neck, which is not present in the homozygote state (nana) [44].

Despite their lack of feathers, Naked Neck chickens are also relatively cold-hardy [45]. Hens weigh between 1.1 and 3 kg, and roosters typically weigh between 1.5 and 3.5 kg. The Naked Neck breed’s annual egg production can reach 138.9, with hens usually laying their first egg at 129 days of age. Furthermore, it has been demonstrated that crossing broiler strains with the naked-neck trait (Na) in tropical climes will result in lower body temperatures, higher body weight gains, better feed conversion ratios, and improved carcass qualities when compared to broilers with normal feathering [46]. However, compared to other chicken breeds, the Naked Neck breed has a slower growth rate, lower survivability, and lower hatchability rate.

### 3.5. Ovambo

Ovambo-land, in Africa’s Northern Namibia, is where Ovambo chickens are originally from [47]. This breed is characterised by its dark colour and smaller size. Considering that they frequently catch and eat mice and baby rats, they are well-known for their aggression and quickness. Moreover, they are able to fly and can use treetop climbing to avoid predators. Another notable quality of the Ovambo breed is its excellent egg output. At 143 days, they usually reach sexual maturity. At 16 weeks, the males weigh approximately 1.7 kg; at 20 weeks, the females weigh 1.32 kg, and at 20 weeks, 1.54 kg [48]. One of the distinguishing characteristics of the Ovambo breed is that it has the highest quantity of dressed meat carcasses among all the South African indigenous chicken breeds [47].

## 4. Challenges During Semen Preservation of Chickens

As the poultry industry grows, there is an increasing demand for cryopreservation technology to store poultry sperm. By enabling the creation of genetic banks that encourage the emergence of unique strains and lines, this technology plays a crucial role in safeguarding the genetic resources of numerous breeds. Despite its significance, sperm cryopreservation has a number of difficulties, the majority of which are related to maintaining sperm fertility and viability during storage. Therefore, the following concepts, the anatomical differences between chickens and mammals, as well as the procedures utilised to preserve avian semen must be comprehended in order to improve the cryopreservation issues in poultry semen.

### 4.1. Differences Amongst Avian and Mammalian Semen

Preserving semen at temperatures below room temperature for extended periods is an effective strategy for managing poultry reproduction. Similar to mammals, two primary methods for avian sperm preservation have been established: liquid storage and cryopreservation [49]. However, the use of preserved semen in poultry production is significantly less common than in mammals [50]. Mammalian and avian species exhibit notable differences in semen volume and concentration. Bulls typically ejaculate between 5 and 8 mL, while roosters produce much less, ranging from 0.1 to 1.5 mL, with an average of about 0.6 mL per collection [14,51]. Avian semen also has a much higher sperm concentration, averaging 6–10 × 10^9^ sperm/mL, compared to 1–2 × 10^9^ sperm/mL in bulls. Excessive dilution of poultry semen can negatively impact sperm function, leading to reduced fertilisation rates and impaired motility and viability [16].

Additionally, the biochemical properties of avian seminal plasma, including carbohydrates, lipids, amino acids, hormones, and proteins, remain poorly understood [52]. Nonetheless, seminal plasma proteins play crucial roles in various cellular functions such as metabolism, immune responses, and antimicrobial defence, which may influence fertility in poultry [36]. The sperm cell plasma membrane is essential for sperm protection and differs between roosters and bulls; roosters have a lower protein-to-phospholipid ratio, which enhances their resistance to cold shock [51,53]. However, rooster sperm membranes contain higher levels of unsaturated fatty acids, making them more susceptible to lipid peroxidation (LPO) and reactive oxygen species during freezing, leading to DNA damage and decreased motility and fertility [16]. Intracellular changes can also result in membrane damage during thawing [51,54]. Enhancing membrane fluidity at lower temperatures may improve cryosurvival [55]. Future research should focus on manipulating the plasma membrane and developing alternative cryoprotectants to enhance the cryopreservation of avian species.

### 4.2. Semen Liquid Storage

Cockerel semen preservation is critical for long-term fertility in chicken breeding, and liquid semen preservation technologies have been intensively investigated [51]. It has been observed that the viability and fertility rate of diluted fowl semen can be maintained for a brief duration of approximately 24 h [56]. Mosenene et al. [14] reported that cockerel sperm, if left undiluted, reduces sperm motility and fertilisation capacity within an hour of collection. Semen extenders are used for either short-term or long-term semen preservation to improve cockerel reproduction and boost breeding rates at a minimal cost through artificial insemination [57]. When comparing liquid-preserved to frozen–thawed semen, liquid-preserved semen has a higher fertilisation rate, which makes artificial insemination (AI) more practical by lowering insemination doses, increasing dose availability, and lowering storage costs [58]. Moreover, it has been reported that the natural in vivo oviductal storage mechanism can retain sperm fertilisation ability for several weeks, whereas in vitro sperm storage only maintains fertilising potential for up to 24 h [58,59]. The variation could be attributed to the selective entry of only morphologically normal and motile sperm into sperm storage tubules, where sperm integrity is retained for future fertilisation [59,60]. Therefore, in order to obtain better results using semen preservation in poultry, the AI techniques in chickens require high-quality sperm, which is deposited near the sperm storage tubules in females [59]. Moreover, the type of extender utilised, as well as storage conditions such as temperature and length, all have an impact on semen preservation success [61].

### 4.3. Semen Cryopreservation

An ideal extender has the following functions: the ability to provide nutrients as a source of energy; the ability to protect against the harmful effects of rapid cooling; and the ability to protect sperm cells during freezing [58]. The semen dilution is performed in order to maximise the number of semen doses, which, in turn, determines the number of females who can be inseminated with a single collection [62]. Antibiotics are added to semen dilution to inhibit the bacterial infestation [63]. The common extenders that are commonly used to cryopreserve cockerel semen are Beltsville poultry semen extender and Lactate ringer egg yolk. Telnoni et al. [64] reported that fertility was high (88%) when Beltsville was used as an extender to cryopreserve white leghorn semen, and Lactate ringer egg yolk resulted in sperm viability rate of 48–49% on liquid preservation during 18 h at 4 °C. The survival of gametes after cryopreservation is significantly influenced by the type and concentration of cryoprotectant (CPA) substances or solutions used prior to freezing [65]. CPAs can be categorised into low- and high-molecular-weight organic compounds that are readily soluble in water and generally exhibit low toxicity, even at higher concentrations. However, the toxic effects of CPAs can vary depending on their type, potentially leading to reductions in various vital and morphological characteristics of sperm, as well as fertility capacity [66]. Consequently, it is essential to evaluate the cell permeability of each cryoprotectant to accurately assess its toxicity. There are two types of cryoprotectants, namely, penetrating and non-penetrating cryoprotectants. Penetrating cryoprotectants are those solvents that dissolve salts and sugars in the cryopreservation medium [67]. Glycerol, dimethyl sulfoxide (DMSO), ethylene glycol, propylene glycol, and demethyformamide are examples of penetrating cryoprotectants [68]. Glycerol is the cryoprotectant mostly used for the cryopreservation of cockerel sperm cells [69]. However, Öztürk et al. [70] reported that glycerol causes detrimental destruction to the sperm cells and increases damage to the DNA of the sperm cell during cryopreservation. Blanch et al. [71] reported that glycerol is a contraceptive for hens when it exceeds 1% and again when hens are intravaginally inseminated [72].

Therefore, it must be reduced before performing artificial insemination by using a centrifuge to reduce its concentration. Dimethylacetamide (DMA) and dimethylformamide (DMF) are the second most commonly used permeable agents for poultry semen cryopreservation (Table 1). The inclusion of DMA or DMF in semen diluents results in a more fluid state of the membranes, facilitating greater dehydration at lower temperatures and enhancing sperm viability after freeze–thaw cycles [16]. Rakha et al. [22]) reported that the addition of 8% DMF to the Indian red junglefowl semen extender improved post-thaw semen quality, including sperm motility, plasma membrane functionality, viability, and acrosome integrity, leading to better fertility in artificial insemination (AI) applications. Similarly, a 6% DMF concentration proved effective for preserving Thai native chicken semen in EK extenders and French straws [73].

While commonly used cryoprotectants can be harmful to poultry sperm viability, non-permeable cryoprotective agents (NP-CPAs) such as polyvinylpyrrolidone (PVP) and polyethylene glycol (PEG) act in the extracellular space. They protect cells by dehydrating the intracellular environment and limiting osmotic swelling during thawing [22]. The addition of NP-CPAs can counteract cryodamage caused by permeable CPAs. At similar concentrations, NP-CPAs tend to be less toxic than their permeable counterparts and provide multiple protective roles, such as inhibiting ice crystal growth and stabilising internal solute concentrations under osmotic stress [51].

Common NP-CPAs used for avian semen cryopreservation include PVP, sucrose, trehalose, raffinose, and, more recently, Ficoll and dextran [22,73,74,75]. Rakha et al. [22] reported that using 6% PVP as the sole cryoprotectant for Indian red junglefowl semen maintained higher sperm cell motility and resulted in better fertility rates during AI compared to diluents containing glycerol. Specifically, the fertility rate with 6% PVP exceeded 70%, surpassing that of glycerol-containing diluents and making PVP a preferable choice for avian semen processing while avoiding the contraceptive effects associated with glycerol.

**Table 1:** Different permeating and non-permeating cryoprotectants used for avian semen cryopreservation.

Species	Cryoprotectant	Diluent	Effect	References
Thai native chicken (Pradu Hang Dam)	DMF 6%	EK extender	Improved sperm motility and yielded lower levels of MDA and high fertility rate	[76]
Chicken	DMA 6%	Tselutin’s extender	Improved sperm motility and resulted in high fertility rate	[72]
Chicken	DMA 6, 9%	Lake extender	Highest proportion of viable and progressive motile sperm recovered after thawing.	[77]
Sandhill crane	DMA 18–26%	Beltsville extender	It improved sperm motility and resulted in high fertility rate	[78]
Chicken	DMF 6% + sucrose 1 mM	Blumberger Hahnen Sperma Verdünner	Increased the vigour motility, membrane integrity, acrosome integrity, and mitochondrial functions of frozen–thawed sperm and high fertility	[73]
Chicken	DMA 6% + trehalose 0.1 M	Lake extender	Improved the recovery rate of progressive motile sperm	[74]
Indian red junglefowl (*Gallus gallus murghi*)	PVP 6%	The red fowl extender (RFE)	Improved motility, membrane integrity, viability, and acrosome integrity and fertility	[22]
Turkey	DMSO + Ficoll 70 1 mM	Tselutin extender	Improved progressive motility and membrane integrity	[75]
Chicken	2% Glycerol + 4% LDL	Beltsville extender	Improved sperm motility after freezing; higher fertility rate	[21]
Japanese quail	11% Glycerol + 0.3% PV	Lake extender	Resulted in successful fertility rates	[79]

In recent years, researchers have increasingly explored the supplementation of cryopreservation mixtures with proteins, such as fetal bovine serum (FBS), bovine serum albumin (BSA), and egg yolk [22,80]. These studies have shown that these proteins can mitigate some of the detrimental effects of cryopreservation, offering promising alternatives for chicken semen extenders. The inclusion of these protein additives enhances the overall viability and functionality of sperm during the freezing and thawing processes, contributing to improved fertility outcomes in artificial insemination applications [80]. On the other hand, egg yolk was also used to preserve avian and mammalian semen. During the freezing process, the egg yolk increases sperm cell viability and serves as a protective agent against temperature-related damage [81]. Research by a number of authors revealed that the fatty acids, phospholipids, and cholesterol found in avian egg yolks, such as those from chicken, quail, pigeons, and ducks, have distinct cryopreservation effects on sperm cells [36,82,83].

According to Kulaksız et al. [84] and Nouri et al. [85], low-density lipoprotein (LDL), cholesterol, and phospholipids found in egg yolk prevent the formation of ice crystals in order to protect the integrity of the sperm cell plasma membrane against cold shock during the cryopreservation process. Furthermore, the low-density lipoprotein from egg yolk has been reported to be a cryoprotectant, which protects sperm cells from cold shock during cryopreservation and also assists in preserving and producing high-quality frozen–thawed sperm [21]. Tshabalala et al. [86] reported that low-density lipoprotein offers protection by decreasing the deleterious effect of seminal plasma protein on sperm cell membranes. Low-density lipoprotein adherence on the surface of the sperm cell plasma membrane restores the loss of phospholipids and induces a temporary change, consequently perverting the rupture of the plasma membrane [87]. Chicken egg yolk has been used successfully as an additive for the cryopreservation of sperm cells because of its availability [36,82]. Bispo et al. [87] reported that low levels of egg yolk (5% and 10%) in semen extender can be used in the cryopreservation of semen. The commercial usage of chicken egg yolk extender is 20%. However, 10% of chicken egg yolk has a positive correlation coefficient in mortality, viability and functional integrity of sperm cell level [88]. Due to variations in egg yolk composition, the possibility of microbiological contamination, and the presence of steroid hormones, the demand for an alternative to egg yolk in freezer extenders has, therefore, increased even more in recent years [89]. Moreover, few studies yielded positive results using chicken egg yolk extender, and they reported the following frozen–thawed sperm cell motility (22.7%, 45%, 25.71% and 29.06%, respectively) [21,22,23,24]. Quail egg yolk has been used by both avian and mammalian species as an extender for cryopreserving semen. Maapola et al. [90] reported that 10% quail egg yolk resulted in sperm cell motility of 39.7% when freezing Venda indigenous chicken semen. Quail egg yolk has the advantage of having a higher concentration of phosphatidylcholine, less phosphatidylethanolamine, and a smaller ratio of polyunsaturated to saturated fatty acids, all of which provide more protection to sperm cells during the cryopreservation process [83].

## 5. Possible Solutions

Optimising cryoprotectant solutions to increase cell survival during freezing and thawing is one potential method for enhancing chicken cryopreservation. The sperm membrane is rich in polyunsaturated fatty acids (PUFAs) [91], which are extremely sensitive to lipid peroxidation (LPO) in the presence of reactive oxygen species (ROS) during storage. This results in decreased sperm motility, DNA damage, mitochondrial malfunction, reduced adenosine triphosphate (ATP) generation, and, eventually, diminished fertility [92]. As a result, increasing antioxidant supplementation and optimising cooling storage processes are critical for ensuring optimum sperm quality throughout preservation.

### 5.1. Antioxidants Supplementation/Addition

Antioxidants are compounds that scavenge or counteract the actions of reactive oxygen species (ROS) [93]. Their primary objectives are to disrupt free radical chain reactions, keep ROS from reacting with cell components, and eliminate the by-products of ROS reactions with cellular macroscopic structures [49,94]. During cryopreservation, some sperm may fail to uptake cryoprotectants due to their small volume, resulting in cell rupture and the release of ROS [51]. Additionally, the cryopreservation process can enhance ROS production in the freezing medium. These ROS can target the bis-allylic methylene group of plasma membrane phospholipids, leading to lipid peroxidation (LPO) [95]. To mitigate the detrimental effects of lipid oxidation during freeze–thaw cycles, it is essential to supplement avian sperm cells with additional antioxidants in the cryopreservation media. Superoxide dismutase (SOD), catalase, glutathione reductase (GR), and glutathione peroxidase (GPx) are among the enzymatic antioxidants present in avian semen [96]. Superoxide anion (O_2_^−^) is broken down by SOD into O_2_ and hydrogen peroxide (H_2_O_2_), and H_2_O_2_ is transformed into O_2_ and H_2_O by catalase [97]. The spermatogenic process and cells require the action of SOD for their protection. According to Partyka et al. [49], equine semen has the highest activity of SOD, whilst roosters have the lowest activity. The tetramer GPx protein is made up of four subunits, each of which has a selenium atom and weighs between 21.5 and 23 kDa. It uses reduced glutathione (GSH) to catalyse the reduction in organic peroxides, especially lipid peroxides, as well as H_2_O_2_ [98]. Studies on birds have shown that the concentrations of GPx in seminal plasma and sperm are greater in goose semen than in chicken semen [49]. The four subunits that make up the tetramer catalase protein each have a molecular weight of 60 kDa. This enzyme is present in sperm cells as well as seminal plasma, and its primary function is to shield cells from hydrogen peroxide’s harmful effects [99]. Information about the presence of catalase in bird semen is scarce. Although semen extenders have long been supplemented with antioxidants (Table 2 and Table 3), natural compounds and small particle antioxidants have been increasingly popular recently. Furthermore, there is a growing body of research suggesting the combination of antioxidants and nanotechnology to improve treatment efficacy and minimise side effects.

### 5.2. New Development in Cryopreservation of Poultry Semen

Improvements in the use of cryoprotective agents (CPAs) are critical for the future development of chicken sperm cryopreservation, as is a better knowledge of the molecular pathways involved. This review emphasises the importance of optimising cryopreservation protocols for avian sperm preservation, noting that routinely employed CPAs can be harmful to chicken sperm viability. As a result, freezing solutions may contain non-permeable cryoprotective substances, such as polymers, carbohydrates, or proteins [51]. These substances aid in modulating intracellular ice crystal formation and stabilising solute concentrations during vitrification [111]. Sugars protect cells by reducing cellular damage and limiting extracellular ice crystal formation through increased extender viscosity, preserving membrane integrity via hydrogen bonding with the phosphate head groups of the lipid bilayer [112]. However, the effects of trehalose and sucrose on post-thaw poultry sperm quality have been inadequately studied, with limited reports available [73,74]. Polymers can also be added to mitigate the toxic effects of CPAs; these non-penetrating agents remain in the extracellular space while reducing the impact of other CPAs [49,51].

Recent research has explored the application of antifreeze proteins (AFPs) in the cryopreservation of poultry sperm [113]. Unlike traditional solutions, these proteins kinetically lower the temperature at which ice crystals form, thereby preventing heat shock [114]. They can modify or inhibit ice crystal growth and prevent recrystallisation, protecting cell membranes from cold-induced damage [115]. Mehdipour et al. [113] investigated Type III antifreeze protein (AFP3) for the cryopreservation of spermatozoa from broiler breeder roosters. Results showed that AFP3 might positively influence sperm cryopreservation, with an optimal concentration of around 1 mg/mL, potentially ranging between 0.1 and 1 mg/mL. However, the high cost of AFP production poses a challenge to its widespread application in cryopreservation [51].

In South Africa, cryopreservation of indigenous poultry semen has demonstrated significant advancements in conserving genetic diversity and enhancing breeding programs. Glycerol is widely employed as a cryoprotectant for freezing poultry semen [14,69,116], although dimethyl sulfoxide (DMSO) has been utilised for the preservation of Venda cockerel semen [90]. Despite this, glycerol continues to be the predominant choice. Therefore, additional research is essential to evaluate the effectiveness of novel cryoprotectants suggested in recent studies. This could enhance the cryopreservation process for South African indigenous poultry breeds and lead to better outcomes.

## 6. Research Gaps

Biologically, the chicken reproductive system differs from that of mammals, making it difficult to plan for their conservation when extinct. For instance, in mammals, semen and oocytes can be harvested and conserved in a frozen state for later use [117]. In contrast, chicken semen can be harvested and cryopreserved, and the frozen–thawed semen survival is still debatable. Moreover, chicken oocytes cannot be harvested and cryopreserved due to the large size and quantity of lipids deposited within the egg [118]. This implies that biological material preservation in chickens is still a one-way process, hindering the success of preservation. Additionally, the chicken reproductive system also includes egg laying and embryo development, which may make them vulnerable to environmental changes and disease infections [6]. While chickens are susceptible to certain infections, their immunological responses are less known than those of mammals, which may impede effective conservation efforts [119]. It is vital to research how these anatomical and physiological distinctions influence breeding success and adaptability to changing settings. Addressing these gaps may assist in maximising conservation practices, improving breeding programs, and establishing better management strategies to ensure the long-term survival of chicken populations across varied ecological contexts [120].

An emerging field of study in chicken conservation is the use of plant extracts to improve poultry health, increase disease resistance, and promote sustainable farming techniques. Plant extracts, such as *turmeric*, *garlic*, and *ginger*, are being studied for their antibacterial, anti-inflammatory, antioxidant, and antifungal properties [121]. These natural substances may minimise the prevalence of common poultry diseases, such as coccidiosis, salmonellosis, and avian influenza, enhancing chicken health and survival. Moreover, it has been shown that plant-based supplements, such as *ashwagandha* and *Moringa oleifera*, increase nutrient intake, lower stress levels, and improve reproductive performance [122,123,124]. Additionally, because of their advantageous properties, plant extracts may be used as semen supplements for semen preservation, such as preserving poultry semen. This is particularly relevant for endangered or uncommon chicken breeds, as they offer a more sustainable and cost-effective option. Therefore, more research is needed to determine the best plant extracts, their suitable dosages, and the most effective applications in order to maximise their benefits for the management and conservation of chicken breeds.

## 7. Conclusions

According to this review, cryopreservation technology for poultry semen is still in its early stages, but it shows great potential for preserving South African indigenous poultry breeds. This developing technology solves the issues raised by geological degradation and decreasing numbers of these distinctive breeds. In addition to offering opportunities for upcoming breeding initiatives and scientific advancements, cryopreservation guarantees the preservation of crucial genetic traits. Therefore, strategies to improve the survival rates of frozen–thawed semen must be developed. The adoption of cryopreservation technology is necessary to ensure the survival of South Africa’s indigenous poultry breeds and their legacy for future generations.

## Figures and Tables

**Table 2:** The use of antioxidants during liquid storage of chicken semen.

Antioxidant	Antioxidant Concentration	Effect	References
Vitamin E (VE)	2, 4, 8, 12, 16, 20 and 40 µg/mL	Improves fertility rate without enhancing motility (8 µg/mL).	[100]
N-acetyl-L-cysteine (NAC)	5 and 15 mM	Enhances kinetic parameters, viability, progressive motility, and sperm motility (15 mM).	[101]
Catalase (CAT)	100 and 300 U/mL	Enhances sperm viability.	[101]
L-carnitine (LC)	0.5, 1, 2, 4 and 8 mM	Enhances motility, viability, and membrane functionality while reducing LPO, ultimately leading to an increased fertility rate (1.2 mM).	[102]
Coenzyme Q10 (CoQ10)	1, 2, 5 and 10 µM	Enhances fertility rate (2.5 µM), decreases lipid peroxidation (5 µM), and enhances sperm motility, progressive motility, membrane functioning, viability, and mitochondrial potential.	[103]

**Table 3:** The use of antioxidants during cryopreservation storage of chicken semen.

Antioxidant	Antioxidant Concentration	Effect	References
Lycopene-loaded liposomes	0.1, 0.2 and 0.3 mM	It reduces malondialdehyde (MDA) levels and apoptosis, increases sperm motility and TAC, and improves fertilisation rates (0 and 2 mM).	[104]
Superoxide dismutase (SOD)	200 U/mL and 50, 100, 200 and 300 U/mL	Improves sperm motility and LPO in sperm cell membranes and shields sperm from apoptosis.	[105]
Mito-TEMPO	0.5, 5, 50 and 500 µM	Enhanced membrane functioning and sperm motility. Moreover, it lowers the level of hydrogen peroxide, DNA fragmentation, and LPO. It improves the 5 and 50 µM fertility rates.	[103]
Quercetin	0.005, 0.01, 0.02 and 0.04 mg/mL	Enhanced viability, mitochondrial activity, acrosome integrity, and sperm motility. Boosts the activities GPx, SOD, and CAT, reduces ROS, MDA, and DNA fragmentation (0.01 mg/mL).	[106]
Melatonin	0.125, 0.25 and 0.5 mg/mL	Promotes the antioxidant activity of enzymes, decreases oxidative stress, and improves sperm motility (0 and 25 mg/mL).	[107]
Ellagic acid-loaded liposomes	0.5, 1 and 2 mM	Enhances mitochondrial activity, membrane functioning, and sperm motility. It increase the activity of SOD at 1 mM, GPx, TAC, and apoptosis.	[104]
Alpha-lipoic acid-loaded lipid nanoparticles	10, 20, 30, 40 and 50 µM	Increases total and progressive motility, encourages viability, and prevents apoptosis. It increases GPx, SOD activity, and TAC (30 µM) while lowering MDA levels.	[108]
Glutathione (GSH)	0.01, 0.02, 0.04, 0.06, 0.08, 0.1, 0.5, 1 and 5 mM	Increases the rate of fertilisation, sperm motility, membrane functioning, and mitochondrial activity. It reduces levels of MDA (2 and 4 mM).	[109]
N-acetyl-L-cysteine (NAC)	5 mM	Enhances sperm motility and mitochondrial activity, strengthens the integrity of the sperm plasma membrane, and helps prevent apoptosis.	[105]
Cysteamine (AET)	0.001, 0.002, 0.004, 0.006, 0.008, 0.05, and 0.1 mM	At 0.001 and 0.004 mM doses, it decreases sperm motility and decreases MDA levels.	[73]
Taurine (T)	1 and 10 mM	Enhances sperm motility and, at 1 mM, preserves the integrity of the plasma membrane, guarding against apoptosis and DNA fragmentation. Furthermore, it lowers LPO.	[110]
Hypotaurine (HT)	1 and 10 mM	Decreases LPO and boosts sperm motility.	[110]

## Data Availability

No new data were created or analyzed in this study. Data sharing is not applicable.
